# Evaluation of household assessment data collected by community health workers in Cape Town, South Africa

**DOI:** 10.4102/safp.v62i1.5168

**Published:** 2020-12-03

**Authors:** Robert Mash, Louiso Du Pisanie, Carla Swart, Ella van der Merwe

**Affiliations:** 1 Division of Family Medicine and Primary Care, Faculty of Medicine and Health Sciences, Stellenbosch University, Cape Town, South Africa

**Keywords:** primary health care, community orientated primary care, community health workers, health information, assessment of health care needs

## Abstract

**Background:**

South Africa has implemented ward-based outreach teams as part of re-engineering primary health care with teams of community health workers (CHWs). In Cape Town, such a community-orientated primary care (COPC) approach was developed at four learning sites. Community health workers registered and assessed the households they were responsible for, but a year later the data were not analysed or converted into useful information. The aim was to analyse the household data and evaluate its contribution to a community diagnosis, its quality and any implications for the performance of CHWs.

**Methods:**

This article used descriptive secondary analysis of household data collected by CHWs at three COPC learning sites in Cape Town (Nomzamo, Eastridge and Mamre).

**Results:**

Data were analysed for 16 852 people from Eastridge, 1338 people from Mamre and 1008 people from Nomzamo. Data were compared in terms of household composition and demographics, type of dwelling, identification of people on treatment for chronic conditions, identification of health risks (e.g. tuberculosis symptoms, tobacco smoking, missed immunisations, missed vitamin A prophylaxis, need for human immunodeficiency virus (HIV) testing or family planning, pregnant or postnatal, and wound care) and for referrals.

**Conclusion:**

Household assessment visits have great potential. Data collected is currently of poor quality, inconsistent or not captured, infrequently analysed and not comprehensive. There is a need to introduce an electronic m-health solution to assist the health information system, to revise the contents of the household assessment form and to ensure that CHWs are competent to identify risks and respond appropriately.

## Introduction

South Africa is committed to a health system built on primary health care (PHC) and has introduced a number of reforms to strengthen this foundation.^1^ These reforms include the employment of district clinical specialist teams to improve maternal and child health, strengthening of school health services and the introduction of ward-based PHC outreach teams. These outreach teams consist of community health workers (CHWs) with a nurse supervisor and are intended to address population health management as a vital component of effective PHC service delivery. Population health management, as defined by the PHC performance initiative, includes local priority setting, community engagement, registration of the population and pro-active population outreach.^2^

Cape Town has implemented ward-based outreach teams within a community-orientated primary care (COPC) approach.^3^ In 2017, the Metropolitan Health Services (MHS) adopted a definition of COPC that was derived from the work of Abramson:^4^

[*A*] continuous process by which primary health care is provided to a defined community on the basis of its assessed health needs, by the planned integration of primary care practice and public health.^3^ (p. 3)

In their framework document, they explained that their intention was to:

[*C*]hange the focus of the health services from only reacting when people become ill enough to present themselves for care, to pro-actively looking at a whole community and addressing the most important challenges together with community members and organisations. This approach means promoting health and more preventative interventions at household and community level, as well as in health facilities.^3^ (p. 3)

This same framework document identified 10 key elements to guide the implementation of COPC (Box 1).^3^ Four learning sites were identified, one in each substructure of the metropole, to learn about the implementation of the new COPC approach. Within these sites, teams of 10–15 CHWs were created around delineated geographic areas and groups of households and linked to the local primary care facility. Each CHW team was led by a professional nurse and employed by a non-profit organisation (NPO) under contract with the MHS. Each primary care facility had a number of CHW teams linked to it in order to cover the whole geographic catchment area and to form a larger PHC team that included the primary care providers (nurse practitioners and doctors) at the facility.

BOX 1 Principles of Metropolitan Health Services’ community-orientated primary care approach.

**Table:** 

Delineation of geographic areas and alignment with primary care facilities and CHW teams
Creating PHC teams of 10–15 CHWs led by a professional nurse, and supported by a nurse practitioner and/or medical doctor
Forming one functional and integrated team across the facility-based and community-based members
Partnership between the MHS and NPOs who employ the CHW teams
Defining a generalist and comprehensive scope of practice for team members
Supporting the teams and COPC approach with a health information system
Engaging with communities around health needs, priorities and assets
Engaging with stakeholders around health needs, priorities and assets
Training the PHC team for COPC
Changing management and communication throughout the system

*Source*: Goliath C, Mash R, Reid S, Mohamed H, Hellenberg D, Perez G. Framework for the implementation of community-orientated primary care in the Cape Town Metro District health services. Cape Town; 2017.^3^

MHS, Metropolitan Health Services; CHW, community health workers; PHC, primary health care; NPO, non-profit organisation; COPC, community-orientated primary care.

The concept of COPC was originally developed in South Africa during the 1940s before being suppressed during the Apartheid era.^5^ The concept was exported and implemented successfully in many other countries such as Brazil, Cuba, Spain, United States of America and Turkey.^6,7^ The family health care team approach in Brazil has had a particularly effective impact on health outcomes.^8^ In Africa, most recent evidence on COPC relates to its implementation, particularly in South Africa, although one study from Kenya has again shown the effectiveness of such an approach.^9^ During the response to the coronavirus disease 2019 (COVID-19) epidemic in Cape Town, the COPC approach to organising the health system has also shown its ability to rapidly adapt to new challenges in PHC.^10,11^ A study commissioned by the department of health in South Africa suggested that there would be a strong case for investment in COPC.^7,12^

In Cape Town, one of the key elements in implementation was to define the scope of practice of the CHWs in a comprehensive and generalist approach. Household registration and assessment was a central part of this approach. Community health workers were expected to visit every household in their area and to gather information on the people living there, the type of dwelling and basic services available, the number of people with chronic conditions and to screen for a variety of health risks such as Tuberculosis (TB), human immunodeficiency virus (HIV), missing immunisations, vitamin A prophylaxis, pregnancy, need for family planning, tobacco smoking and wound care. People with such risks could be counselled or referred to appropriate services.

Whilst many studies have investigated factors impacting on the implementation of ward-based outreach teams,^9^ there has been little focus on the quality and usefulness of household assessment data collected by CHWs. This study, therefore, aimed to look at the household assessment data collected over a period of 1-year at the learning sites. At all these learning sites data had been collected on paper and in some cases captured by the NPO on electronic spreadsheets. None of the sites had actually analysed their data. Key objectives were to evaluate these data in terms of the quality of the data collected, the contribution of these data to a community diagnosis and understanding of local priority health needs, and the performance of the CHWs in identifying individuals with health issues and assisting them.

## Methods

### Study design

The study performed a secondary descriptive analysis of data already collected by CHWs at COPC learning sites in Cape Town.

### Setting

The Cape Town MHS is divided into four substructures and one community was selected as a COPC learning site by the MHS in each substructure. Each learning site had at least one primary care facility and one NPO that employed the CHW teams:

- Northern-Tygerberg substructure: Bishop Lavis community with Bishop Lavis Community Health Centre (CHC) and Caring Network (NPO)
- Klipfontein-Mitchells Plain substructure: Eastridge community with Mitchells Plain CHC and Arisen Woman (NPO)
- Southern-Western substructure: Mamre community with Mamre Community Day Centre (CDC) and the TB-HIV Care Association (NPO)
- Eastern-Khayelitsha sub-substructure: Nomzamo community with Nomzamo CDC, Ikwezi clinic and Masincedane (NPO).

The Bishop Lavis learning site was excluded from this study as household data had recently been analysed by a community health registrar in an unpublished report.

**FIGURE 1:**
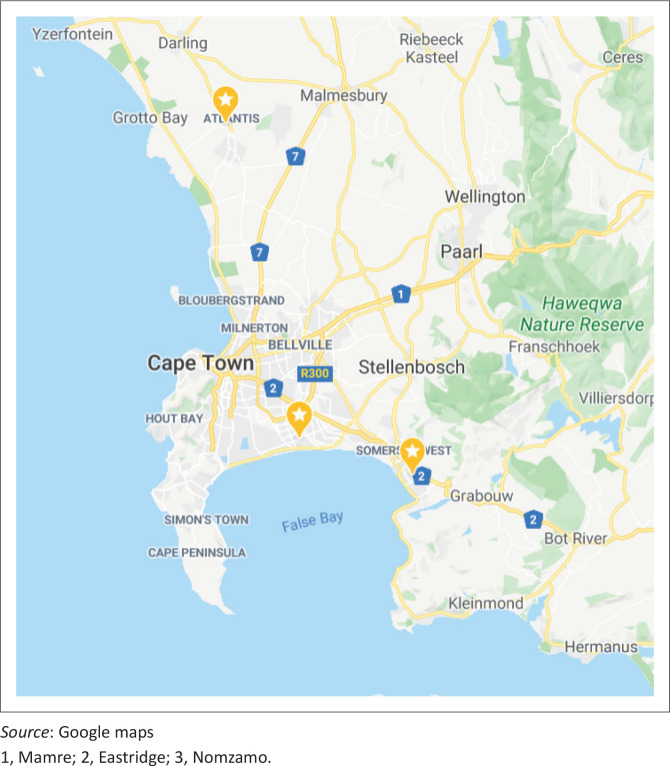
Location of learning sites in Cape Town.

Eastridge is a suburb on the Cape Flats with 5953 households and 28 482 people in the 2011 census (Figure 1).^13^ It forms part of Mitchells Plain, which was built as a township in the 1970s to house so-called ‘coloured’ people who were forcefully removed from other parts of Cape Town.

Mamre is a small rural town on the edge of the Cape Town metropole with a population estimated at 9048 in 2226 households.^13^ It was originally founded by Moravian missionaries in 1808.

Nomzamo is situated on the edge of Cape Town in Somerset West with an estimated population of 60 528 in 19 520 households.^13^ Historically, Nomzamo was an area designated for black Africans under apartheid and was initially for single male workers living in hostels. In the 1990s, the population expanded with informal settlements.

### Sampling and data collection

Data had already been collected by CHWs, who were meant to visit every household in their designated geographic area. In Mamre and Eastridge, the data were captured by the NPO on an electronic spreadsheet and all the data from a 1-year period were included in the analysis. In Nomzamo, the data were still on the paper-based household assessment forms and stored in a cupboard. The NPO had an electronic list of 1500 households that had been visited and this was used to select a random sample of forms from a 1-year period. All data were from the 2017 to 2018 period.

### Data analysis

Data were prepared in Excel spreadsheets from each site and checked for errors or omissions. Data were then imported to the Statistical Package for Social Sciences (SPSS) version 26 for descriptive analysis. Categorical data were reported as frequencies and percentages, whilst numerical data were reported as means and standard deviations or medians and interquartile range depending on the distribution of the data.

The learning sites differed in the way that they had captured the data. For example, Eastridge captured the data per household, whereas the other sites captured the data per individual. Mamre captured age as less than 5 years or more than 5 years, whilst other sites captured the actual age. These differences made it difficult to analyse the data set as a whole.

### Ethical consideration

Ethical approval to conduct the study was obtained from the Health Research Ethics Committee, Stellenbosch University on 16 November 2018 for Mamre, 03 October 2018 for Nomzamo, 01 February 2019 for Eastridge. Ethical clearance numbers were U18/10/033 for Mamre sub-study, U18/07/025 for Nomzamo and U19/01/005 for Eastridge.

## Results

In Eastridge, data were analysed from 3564 households with 16 852 individuals, in Mamre from 1338 households with 3895 individuals and from Nomzamo 321 households with 1008 individuals. In Eastridge, data were collected at the household level and only some items could be extrapolated to individuals. The data sets were otherwise very complete for the demographic and housing information. Table 1 presents the findings in terms of the population demographics and characteristics of the households.

**TABLE 1a:** Demographics of population and characteristics of households.

Variables	Eastridge *N* = 3564†		Mamre *N* = 3895		Nomzamo *N* = 1008	
	*n*	%	*n*	%	*n*	%
**Sex**						
Male	7925	47.1	1688	43.3	432	45.8
Female	8886	52.9	2207	56.7	512	54.2
**Age (years)**						
< 5	1681	10.0	216	5.5	126	13.2
5–18	4280	25.6	3676	94.5	237	24.9
> 18	10 789	64.4			590	61.9
**Social services**						
Social grant	1982	55.6	883	67.5	200	22.4
**Type dwelling**						
Formal	2522	71.3	3362	86.4	183	19.1
Formal in backyard	892	25.2	437	11.2	362	37.9
Informal in backyard	87	2.5	46	1.2	141	14.7
Informal	38	1.1	47	1.2	270	28.2
**Basic services**						
Electricity	3530	99.7	3805	99.3	829	82.2
**Water access**						
In house water	3335	94.4	3675	97.5	392	41.8
In yard	13	0.4	66	1.8	275	29.3
Communal < 200 m	185	5.2	27	0.7	249	26.5
Communal > 200 m	0	0.0	NA	-	22	2.3
**Toilet access**						
Flush toilet available	3336	94.2	3792	99.4	813	90.0
In house	3403	96.3	3726	99.3	333	40.8
In yard	124	3.5	26	0.7	209	25.6
Outside yard	7	0.2	NA	-	275	33.7

† , *N* = 16852 for people in calculation of age and sex. Other variables use *N* = 3564 for households.

NA, not available; IQR, interquartile range.

**TABLE 1b:** Demographics of population and characteristics of households.

Household members	Eastridge *N* = 3564		Mamre *N* = 3895		Nomzamo *N* = 1008	
	Median	IQR	Median	IQR	Median	IQR
People per household	4.0	3.0–6.0	NA	-	4.0	3.0–6.0
Rooms per household	5.0	3.0–5.0	5.0	3.0–6.0	2.0	1.0–3.0

Mamre appeared to be an older population on social grants (probably pensions), with fewer children, in a community with almost all formal housing and good access to water and flush toilets in the house. Eastridge also had a substantial proportion of households receiving a social grant, mostly formal dwellings and good access to water and flush toilets. Nomzamo on the other hand was a much poorer community with a slightly younger population profile, smaller houses with more crowding, relatively fewer social grants (probably less pensions), much higher levels of informal housing and much less access to running water and flush toilets in the house.

Table 2 shows the percentage of the population that was identified as being on medication for chronic conditions. Again, Eastridge is reported differently as the proportion of households with a person on medication. The accuracy of this information was significantly limited by the amount of missing data, where it was not clear if the CHW had screened the household. Nevertheless, trends are clear in the sense that HIV and TB were more prevalent in Nomzamo, whilst non-communicable diseases were a much larger problem in Eastridge and Mamre.

**TABLE 2:** Proportion of population identified as taking medication for chronic conditions.

Variable	Eastridge *N* = 3564†		Mamre *N* = 3895		Nomzamo *N* = 1008	
	*n*	%	*n*	%	*n*	%
HIV	41	1.1	10	0.3	97	9.6
Tuberculosis	50	1.4	10	0.3	62	6.2
Hypertensive	1399	39.3	893	22.9	40	4.0
Diabetes mellitus	754	21.2	322	8.3	30	3.0
Asthma	510	14.3	86	2.2	30	3.0
Psychiatric	117	3.3	26	0.7	10	1.0

† , households.

HIV, human immunodeficiency virus.

Table 3 shows the number of people identified as having TB symptoms during the household assessments. The numbers are generally very small and again the information was limited by large amounts of missing data, where it was not clear if screening was performed or not.

**TABLE 3:** Proportion of population identified as having tuberculosis symptoms.

TB symptoms	Eastridge *N* = 3564†		Mamre *N* = 3895		Nomzamo *N* = 1008	
	*n*	%	*n*	%	*n*	%
Cough	14	0.4	7	0.2	18	1.8
Night sweats	11	0.3	8	0.2	18	1.8
Weight loss	9	0.3	4	0.1	29	2.9
Loss of appetite	3	0.1	1	0.0	16	1.6
Fever	31	0.9	3	0.1	21	2.1

† , households.

TB, tuberculosis.

Table 4 shows the proportion of the population identified as having a number of specific health needs. Mamre’s data suggested a large number of children were not immunised or received vitamin A prophylaxis, whilst in Nomzamo very small numbers of children were identified. This reflects a substantial amount of missing data where it is not clear if children were actually screened. At Eastridge, a substantial portion of data were identified at household level and suggested high rates of immunisation and children receiving vitamin A prophylaxis. Very small numbers of women needing assistance with pregnancy and family planning were identified. The majority of households in Eastridge had a member that smoked tobacco, whilst 27% of people in Mamre were tobacco smokers. The lower proportion in Nomzamo reflects a large amount of missing data, where it is not known if people were asked about smoking.

**TABLE 4:** Proportion of population identified as having a health risk.

Variable	Eastridge *N* = 3564†		Mamre *N* = 3895		Nomzamo *N* = 1008	
	*n*	%	*n*	%	*n*	%
**Child health (< 5 years)**						
Immunisations not up to date	4/1288	0.3	117/216	54.2	14/126	11.1
Vitamin A not up to date	11/1288	0.9	117/216	54.2	16/126	12.7
**Women’s health**						
Post-natal women	1	0.2	2	0.1	4	0.4
Pregnant women	47	1.3	3	0.1	13	1.3
Needing family planning	NA	-	3	0.1	15	1.5
**Other health risks**						
Tobacco smokers	2105	59.0	1037	26.6	92	9.1
Needing HIV test	10	0.3	29	0.8	39	3.9
Needing wound care	4	0.1	1	-	8	0.8

† , denominator is households.

NA, not available; HIV, human immunodeficiency virus.

In Mamre, only 6 out of 272 (2.2%) of those identified as having a health risk had a record of referral in the household assessment form. In Eastridge, a different referral system was used, which was not captured on the electronic database. In Nomzamo, 45 out of 162 (27.8%) of those identified were recorded as referred.

## Discussion

At all learning sites, CHWs had visited a large number of households and completed household registrations and assessments during the first year of the COPC initiative. There was thus a huge potential for useful information. The findings demonstrated significant issues with the quality of the data and possibly with the performance of the CHWs. These issues impaired the usefulness of the data and the ability to accurately identify health needs of the communities. The findings also illustrate the strengths and weaknesses of the data in terms of giving a comprehensive view of health needs in these communities.

In all learning sites, there was a problem with missing data for many of the items. It was impossible to determine if the CHWs had asked everyone the relevant questions and only recorded positive responses or whether the questions had not been asked at all. With many items there were very few people identified (e.g. TB symptoms, missing immunisations and chronic medication), suggesting that CHWs had not consistently collected data from all household members. The household assessment form itself did not always require a definite answer and encouraged CHWs to only record a ‘yes’ answer by ticking. It is also possible that the household members who assisted the CHWs were not able to answer all of the questions for other household members who were absent. The extremely low number of referrals suggested that CHWs were using other mechanisms to record referrals and were not completing this aspect of the household assessment to save time and avoid duplication of tasks. Nevertheless, the low number of referrals raised a question as to whether CHWs were competently recognising health risks and responding to them appropriately. This question could not be answered from this data.

The need to conduct a study of this nature to analyse data that had been collected at least a year ago illustrates the flawed nature of the health information system for COPC and household assessments. None of the NPOs had the capacity to analyse the data themselves and in one case were just storing the forms in a cupboard. In addition, none of the data had been captured or analysed by the department of health. It was clear that an electronic m-health solution would potentially solve many of the problems. If data were collected via cell phone, then CHWs could be prompted to enter data more completely and accurately. Data would automatically be captured in a standardised way and analysed in terms of pre-determined indicators. Results could be immediately available to users at different parts of the system from the NPOs, primary care facilities to managers at substructure and district level. Data could be easily entered into the provincial data centre and combined with other individualised data to contribute to a personal electronic medical record. A locally developed m-health solution called Catch and Match was introduced at two of the learning centres during the implementation of COPC but has not yet delivered on its full potential.

The household assessment form does not provide a comprehensive view of the health risks or the CHWs’ scope of practice. In terms of health promotion and disease prevention, there is a focus on TB and HIV, but little attention to non-communicable diseases, other than tobacco smoking. Local research on CHWs has shown the ability to assess cardiovascular disease risk using a non-laboratory approach.^14^ For example, identifying people with a high risk (> 20% of an event in the next 10 years) could lead to useful counselling and referral for further assessment. Apart from wound care there is no identification of the need for physical care. For example, people with disabilities, such as stroke survivors and their caregivers, may need substantial help,^15^ as well as people needing palliative care.^16^ In terms of child health, there is no space for assessment of growth and malnutrition. Injuries and trauma are also a major contributor to the burden of disease^17^ and there is no focus on suspected alcohol or substance abuse, intimate partner violence, other forms of abuse or disability as a consequence of injuries. Maternal and women’s health is covered to some extent but need for cervical and breast cancer screening could be another useful addition. Finally, there is no prompting to consider social services and social grants are not disaggregated into different types (e.g. old age pension, disability and child support). It is clear that as a more comprehensive scope of practice is clarified that the household assessment form will need to be revised and aligned with this. Some of the missing data may be sensitive and the CHW would have to gain the trust of household members and maintain confidentiality.

Ultimately, the purpose of the CHWs is to assist the population at risk to live a healthier life. In the household, they need to have generalist skills to enter, form a relationship, identify any health risks, and respond helpfully and appropriately. There is a risk that the household assessment becomes an administrative exercise in obtaining data or filling out a form for its own sake. Completion of the form is used to evaluate CHW performance. Community health workers must retain a person centred approach to their work and use the household assessment form as a prompt and a guide to useful action in service of the community and household members.

### Limitations

The study was reliant on CHWs to collect the data and, therefore, the representativeness and quality of the data were out of the researcher’s control. The analysis however shed light on issues with the type and quality of data and the performance of the CHWs. Data were also captured in slightly different ways by NPOs making inferential analysis difficult. Data have, however, been presented as far as possible in a comparable manner.

### Recommendations

This study supports the need for:

- Introduction of an electronic m-health solution to ensure household assessment data are reliably collected, captured and analysed. In addition, information derived from the data should be made accessible downstream (to CHWs, professional nurses, primary care facilities and community health forums) and upstream (to substructures, district, province and national levels).
- Review of the contents of the household assessment form to align with the CHWs’ new scope of practice and the need to provide a comprehensive overview of community health needs.
- Review of the competence of CHWs to identify health risks and to act appropriately. Any learning needs should be addressed through in-service training and by the professional nurses leading the CHW teams.

## Conclusion

Household assessment visits have great potential to identify individuals at risk and to help appropriately as well as to contribute to a community diagnosis that identifies local health priorities. Data collected is currently of poor quality, inconsistent or not captured, infrequently analysed and not comprehensive. There is a need to introduce an electronic m-health solution to assist the health information system, to revise the contents of the household assessment form and to ensure that CHWs are competent to identify risks and respond appropriately.
